# The physicochemical and biomechanical profile of forsterite and its osteogenic potential of mesenchymal stromal cells

**DOI:** 10.1371/journal.pone.0214212

**Published:** 2019-03-27

**Authors:** Genasan Krishnamurithy, Saktiswaren Mohan, Noor Azlin Yahya, Azura Mansor, Malliga Raman Murali, Hanumantha Rao Balaji Raghavendran, Rajan Choudhary, Swamiappan Sasikumar, Tunku Kamarul

**Affiliations:** 1 Tissue Engineering Group (TEG), Department of Orthopaedic Surgery (NOCERAL), Faculty of Medicine, University of Malaya, Kuala Lumpur, Malaysia; 2 Musculoskeletal Research Group, Department of Molecular and Clinical Cancer Medicine, Institute of Translational Medicine, Liverpool, United Kingdom; 3 Faculty of Dentistry, University of Malaya, Kuala Lumpur, Malaysia; 4 Department of Chemistry, School of Advanced Sciences, Vellore Institute of Technology, Vellore, Tamil Nadu, India; Università degli Studi della Campania, ITALY

## Abstract

It has been demonstrated that nanocrystalline forsterite powder synthesised using urea as a fuel in sol-gel combustion method had produced a pure forsterite (FU) and possessed superior bioactive characteristics such as bone apatite formation and antibacterial properties. In the present study, 3D-scaffold was fabricated using nanocrystalline forsterite powder in polymer sponge method. The FU scaffold was used in investigating the physicochemical, biomechanics, cell attachment, *in vitro* biocompatibility and osteogenic differentiation properties. For physicochemical characterisation, Fourier-transform infrared spectroscopy (FTIR), Energy dispersive X-ray (EDX), X-ray diffraction (XRD), Raman spectroscopy, X-ray photoemission spectrometer (XPS) and Brunauer-Emmett-Teller (BET) were used. FTIR, EDX, XRD peaks and Raman spectroscopy demonstrated correlating to FU. The XPS confirmed the surface chemistry associating to FU. The BET revealed FU scaffold surface area of 12.67 m^2^/g and total pore size of 0.03 cm^3^/g. Compressive strength of the FU scaffold was found to be 27.18 ± 13.4 MPa. The human bone marrow derived mesenchymal stromal cells (hBMSCs) characterisation prior to perform seeding on FU scaffold verified the stromal cell phenotypic and lineage commitments. SEM, confocal images and presto blue viability assay suggested good cell attachment and proliferation of hBMSCs on FU scaffold and comparable to a commercial bone substitutes (cBS). Osteogenic proteins and gene expression from day 7 onward indicated FU scaffold had a significant osteogenic potential (*p*<0.05), when compared with day 1 as well as between FU and cBS. These findings suggest that FU scaffold has a greater potential for use in orthopaedic and/or orthodontic applications.

## Introduction

Bio-ceramics are one of the ultimate substitutes for restoring bone defects, which may either have the role of providing support, filling hollow spaces or enhancing the biological activity of *in situ* bone tissue [1]. The inherent characteristics such as biocompatibility, bioinert and durability of these materials allow them to be as a primary choice in engineering orthopaedic or orthodontic implants [2, 3]. To date, bio-ceramics such as alumina, zirconia, silicate, and phosphate ceramics have been gaining much attention among the ceramists [4]. However, certain limitations associated with these bio-ceramics such as the intrinsic brittleness, poor wear resistance and fracture toughness restrict their wide-ranging applications for the treatment of bone defects [5]. These challenges can be overcome by exploring silicate based bioactive ceramics for bone tissue engineering. The superior fracture toughness, excellent wear resistance and the osteogenic inducing characteristics such osteoinductive and osteoconductive properties indicate silicate ceramics can be a potential biomaterial for hard tissue regeneration [6, 7]. Recently, Shie *et al*. (2017) found that silicates have a potential to form direct chemical bonding with local tissue, thus they support osteogenesis and angiogenesis [8].

Bioactive silicates including wollastonite, larnite, diopside, akermanite, and merwinite, bredigite have been characterised for their physicochemical properties [9, 10]. However, the feasibility of these materials in studies aiming for bone tissue engineering application yet to be fully understood, therefore, these materials require a detailed investigation. Nonetheless, the investigation on Mg and Si based silicates, primarily the forsterite (Mg_2_SiO_4_) has surpassed many other silicate based bio-ceramics [11]. Recently, the incorporation of forsterite with poly(lactic acid) using 3D printing and electrospinning technique produced promising material that could be attributed as a choice in bone tissue engineering [12]. In another study, nanostructured forsterite coated on stainless steel implants promoted their biocompatibility characteristic [13]. Moreover, forsterite was found retaining excellent fracture toughness when compared with hydroxyapatite or bio-glass [14, 15]. These superior properties, thus, recommend that forsterite must be explored thoroughly to recognise its applicability in the field of bone tissue engineering.

Earlier, we successfully prepared nanocrystalline forsterite powder using urea as a fuel in sol-gel combustion method [16]. Our preliminary investigation on nanocrystalline forsterite bioactivity role demonstrated an excellent appetite formation in simulated body fluid (SBF) and anti-microbial activity such as inhibiting the growth of Staphylococcus Aureus (*S*. *aureus*). However, to employ a silicate for bone tissue engineering application, it is highly essential to confirm their biocompatibility and osteogenic activities *in vitro* condition prior to be used *in vivo* testing. In this respect, the osteoblast precursor, which is mesenchymal stromal cells (MSCs) have become superior [17]. MSCs have an intrinsic characteristic to functionally commit to osteogenic lineage for bone formation [18, 19]. In pathophysiological condition such bone fracture, MSCs derived osteoblasts/osteocytes collaborate with osteoclast to conduct bone remodelling and mineralisation [20]. Since nanocrystalline forsterite has emerged as a potential candidate for bone tissue engineering, its application as a scaffold to support MSCs adhesion and osteogenic differentiation needs further validation. Therefore a study was designed to investigate the characteristic of nanocrystalline forsterite in supporting human bone marrow derived MSCs (hBMSCs) adhesion and osteogenic lineage commitments *in vitro*.

## Materials and methods

### Fabrication of forsterite (FU) scaffold

FU was prepared by sol-gel combustion route by using urea as a fuel. The detailed procedure adopted for the preparation of FU powder is explained in our recent publication [21]. The FU scaffold for cellular studies was fabricated using polymer sponge method. Briefly, 10% polyvinyl alcohol (PVA) was added dropwise (2–3) to finely grinded FU powder (500 mg) and pelletised in the form of circular scaffolds. These scaffolds were heated in furnace at 800°C for 2 h, to burnout polymer content and generate porosity in the samples.

### Physicochemical characterisation

Energy dispersive X-ray analysis was studied using EDX-System (INCA Energy 200, Oxford Instruments). The energy of the X-rays emitted from the samples was measured using an energy-dispersive spectrometer and the corresponding EDX spectrum was plotted using Micro-analysis suite software (Version 4.05-Oxford Instruments). The Bragg peaks XRD patterns of FU scaffold were recorded on a D8 Advance X-Ray diffractometer (Bruker-AXS, USA). The diffractometer was operated using Ni-filtered monochromatized CuKα radiation (λ = 1.54056Ǻ) at 40kV, and 40mA at 25 ºC with a scanning rate of 0.1 deg s^-1^. Bragg peak diffraction patterns were plotted at the range of 10 to 80 2θº. For Raman spectroscopy (Renishaw Invia, UK), the Raman spectra were collected with laser excitation at 514 nm and an acquisition range from 200 to 1000 cm^-1^. An X-ray photoelectron spectroscopy (XPS, ULVAC-PHI Quantera II) with C Kα (hν ¼ 278–298 eV), O Kα (hν ¼ 523–543 eV), Mg Kα (hν ¼ 84–104 eV), Si Kα (hν ¼ 94–144 eV), Ar Kα (hν ¼ 235–255 eV), Ca Kα (hν ¼ 341–361 eV) and Hg Kα (hν ¼ 95–105 eV) X-ray sources was utilized for FU XPS signal acquisition. The Brunauer−Emmett−Teller (BET-Autosorb iQ2) specific surface area of the FU scaffold was measured from the nitrogen adsorption-desorption isotherms.

### Biomechanical property

Unconfined isostatic compression test was carried out at a loading speed of 10 mm/min between parallel steel plates using uniaxial compression machine (Instron model 3365, USA). Cylindrical FU scaffolds (n = 3) were prepared with an average dimension of ~10 mm (diameter) and ~5 mm (height). Stress–strain curve following compression of the FU scaffold was generated using Instron software version Blue Hills 2.

### Cell culture

#### hBMSCs isolation & culture

The authors have obtained University of Malaya Medical Centre Ethics Committee approval (Ethics No: 967.10) and followed the principles outlined in the Declaration of Helsinki for human experimental investigation. Human Bone marrow aspirates were obtained from subjects (50–70 years old) undergoing total knee/hip replacement. Informed consent was obtained prior to acquiring samples. The hBMSCs isolation methods were adopted from our published article [4]. Briefly, bone marrow mononucleated cells (MNCs) were purified using standard Ficoll-Pague gradient centrifugation (density 1.073 g/mL) according to manufacturer’s instruction (GE Healthcare Bio-Sciences, USA). The density gradient centrifugation was performed at 2200 rpm for 25 min. The middle layer containing MNCs was isolated and washed three-times with PBS (1X) (Gibco, Invitrogen, USA). The MNCs were then suspended in culture medium DMEM-LG (Gibco) containing 100 U/mL of penicillin and 100 μg/mL of streptomycin supplemented with FBS (Gibco). Cell number and viability were enumerated using the trypan blue exclusion method. About 1 X 10^6^ cells were seeded onto T-75 culture flask and incubated at 37°C in 5% CO_2_ with 95% humidity. For subsequent passaging, the cells in passage-0 (P_0_) were washed with PBS (1X) and then incubated in trypsin (TrypLE, Gibco) for 3 min in a CO_2_ incubator at 37°C until complete cell detachment observed. Harvested P_0_ cells were sub-cultured into passage-1 (P_1_) and the culture medium was changed on every 3 days interval.

### hBMSCs characterisation

#### Plastic-adherent and morphology analysis

Phase contrast microscopy (PCM) was performed using inverted light microscopy (Nikon, Japan) to determine passage-1 (P_1_) morphology of cells cultured on the T75 plastic flasks. The cells were observed at X10/0.25 objective lens (Olympus, USA) and images captured using Xcam-α digital camera (Olympus) up to three image fields/experimental group.

#### Flow cytometry analysis

Adherent stromal cells 1 X 10^6^ cells/mL were trypsinised at P_1_ and washed twice with DMEM in 15 mL centrifuge tube. The cell pellet was re-suspended in 100 μL of PBS (1X) and equally distributed into two flow cytometry tubes labelled either as unstained and stained tubes. For primary monoclonal antibody staining, 5 μL of CD34-PE, CD105-PERCP 5.5, CD73-FITC, CD90-PE-Cy7, CD 45-APC-H7 and CD 44-APC were added in staining tube. The isotype controls for the selected primary monoclonal antibody were used to differentiate non-specific background signal from specific antibody signal (S1 Fig). The tubes were incubated on ice for 30 min in the dark. After incubation, cells were washed in 2 mL of PBS (1X) and centrifuged at 1200 rpm for 5 min to remove the unbound antibodies. The pellet was then re-suspended in 500 μL of PBS (1X) and acquired in BD FACSCanto II flow cytometry. Data was analysed using BD FACSDIVA Software and cell gating performed using previously established method [22].

#### Tri-lineage differentiation

P_1_ hBMSCs were seeded on the four-chamber slide at seeding density of 1 X 10^3^ cells/cm^2^ in all wells and incubated in DMEM with 10% FBS at 37 ºC in 5% CO_2_ with 95% humidity until the cells become 80% confluency. The cells on the chamber slide were then treated either with DMEM, osteogenic or adipogenic differentiation medium (StemPro, Invitrogen) and medium changed every 3 days interval. On the day 14, the chamber slide seeded cells were washed with PBS (1X) and fixed with 4% formaldehyde. The DMEM treated cells were stained with 1:100 diluted mouse CD44 primary antibody solution and counterstained with 1:100 diluted rat secondary antibody. The cells treated with osteogenic and adipogenic medium were stained with Alizarin-red S and Oil-red O staining, respectively. For chondrogenic differentiation, cell-pellet at a density of 1 X 10^6^ cells/mL was treated with chondrogenic media until day 14. The cell-pellet was then fixed with 4% formaldehyde and subjected to waxing, sectioning, mounting onto slides and staining with Safranin-O. Imaging was performed using bright field microscope with CFI E Plan Achromat X40/0.65 objective lens (Nikon Eclipse E200, USA) and 4.65 x 4.65 μm pixel digital camera (Infinity 2, Lumenera Corp., USA).

### Cell seeding

For sterilisation, FU scaffolds were sent for 25 kGy gamma irradiation (Nuclear Agency of Malaysia). The cell seeding was performed using a published method [23]. Briefly, hBMSCs were enzymatically detached using 3 mL of trypsin after reaching 80% of confluence at P_1_. A cell suspension was prepared and seeded onto the FU scaffolds and commercial bone substitute (cBS, CERAFORM, France), which was a baseline control, in low attachment twelve-well plate in drop-wise manner and in six-well plate (monolayer) at the density of 1 X 10^6^ cells/mL. The cell-seeded FU scaffolds and cBS were cultured using osteogenic medium (StemPro, Invitrogen). Medium were collected on day 1, 7, 14 and 21 from the cell-FU scaffold group and cell-cBS group stored in -20 ºC for alkaline phosphatase and osteocalcin analysis.

### Cell attachment analysis

#### Scanning electron microscopy analysis

Scanning electron microscopy (SEM) analysis was performed to observe surface topography of hBMSCs seeded on FU scaffolds (n = 3) and cBS (n = 3). The samples at day 14 were fixed overnight in 4% glutaraldehyde in 0.1M cacodylate buffer and post-fixed for 1 h in 1% aqueous osmium tetroxide. These samples were washed with three consecutive steps in distilled water before being dehydrated through a graded ethanol series (50, 70, 80, 90, 95 and 100%). The samples were subsequently dried at a critical point using critical point drier (Bal Tec, CPD030). The samples were mounted on aluminium stub and sputter coated with gold before being examined using a digital scanning electron microscope (JSM 6400; JEOL, Tokyo, Japan).

#### Confocal laser microscopy analysis

Confocal laser scanning microscopy (CLSM) analysis was performed to determine the cell density and morphology on the surface of the material. The hBMSCs seeded on FU scaffolds (n = 3) and cBS (n = 3) on Day 14 were stained using Alexa Fluor 488 phallotoxins F-actin and counterstained with Hoechst 33342 nucleic acid dye (Life Technologies, Invitrogen). FU scaffold and cBS without cells was used as a control. Samples were stained according to the protocol provided by manufacturer. After 20 min of incubation, the samples were washed with PBS (1X). All images were acquired using Leica TCS SP8 X inverted confocal microscope with X20/0.40 NA objective lens and 2X-zoom (Leica) and analysed using Leica Application Suite X imaging software (Leica LAS X, UK).

### Cell proliferation analysis

Cell proliferation in FU scaffolds (n = 3) and cBS (n = 3) was assessed using the colorimetric indicator Presto Blue (PB) cell proliferation/viability assay (Gibco). The assay was performed based on the PB reduction on day 1, 7 and 14. PB was directly added into the media in all preparation at final concentration of 10% and incubated for 10 h. After incubation, 100 μL of medium from each sample was transferred into a 96-well plate in duplicates. PB added to hBMSCs monolayer was served as a baseline control and samples without cells served as a blank. Absorbance in each well was measured at 570 and 600 nm (reference wavelength) using a microplate reader (Epoch, USA). The corrected absorbance readings were calculated by subtracting the individual reference wavelength from respective measured wavelength and presented as mean ± standard deviation (SD).

### Immunocytochemistry (ICC)

For immunofluorescence staining, the cells seeded FU scaffolds (n = 3) and cBS (n = 3) on day 1 and 14 were fixed with 4% (w/v) paraformaldehyde (PFA) (Sigma, USA) for 15 min at room temperature (RT) and stained as described previously [24]. For primary antibodies, BMP2 (anti-BMP2 antibody [IgG]) (1 μg/mL; Abcam, UK), Type-I collagen (Col1) (anti-Collagen 1 antibody [IgG1]) (1/1000; Abcam), Osterix (OSX) (anti-Sp7/Osterix antibody [IgG]) (1/1000; Abcam), RUNX2 (anti-RUNX2 antibody [IgG2a]) (10 μg/mL; Abcam) and Osteopontin (OPN) (anti-Osteopontin antibody [IgG2a]) (1/1000; Abcam) were used. The Chicken polyclonal secondary antibody conjugated with Alexa Fluor 647 (1:500; Abcam) was used and counterstained with Hoechst 33342 nucleic acid staining. The fluorescence signals were observed using Leica TCS SP8 X inverted confocal microscope with X20/0.40 NA objective lens and 2X-zoom (Leica) and analysed using Leica Application Suite X imaging software (Leica LAS X, UK). For total cellular fluorescence calculation, the confocal images were further processed using Image-J analysis software (IJ 151j/Java 1.8.2-64-bit, NIH, USA). Three random regions of interest (ROIs) were assigned for each interrogation and the corrected total cell fluorescence (CTCF) was calculated using the following equation: CTCF integrated density—(area of selected ROI × fluorescence of background reading) [4]. Data were presented as mean ± SD.

### Biochemical analysis

#### Alkaline phosphatase assay

ALP activity was measured on cell-scaffold (FU/cBS) culture media collected at day 1, 7, 14 and 21 using an ALP colorimetric assay kit (BioVision, USA). A 50 μL of media from cells seeded FU scaffolds (n = 4) and cBS (n = 4) in duplicates was mixed with 30 μL of p-NPP substrate. The aspirates were then added with assay buffer solution to make a final volume of 130 μL and incubated for 60 min at 25 ºC, protected from light. Samples background control was also prepared using the same method as described above. A 20 μL of stop solution was added to all background controls before 60 min incubation period, except for all aspirates which were added upon completion of the incubation period. The absorbance of the aspirates and background controls was measured at a wavelength of 405 nm using a microplate reader (Epoch). The optical density values for each calibrator against the corresponding concentration of ALP were plotted onto an excel sheet to produce a standard curve. The ALP concentration in aspirates was extrapolated using linear equation calculated from this standard curve.

#### Osteocalcin assay

Osteocalcin (OC) assay was performed on cell culture media collected from cells seeded FU scaffolds (n = 4) and cBS (n = 4) using human OC-ELISA assay kit (IBL International, Germany) on day 1, 7, 14 and 21. Wells from the primary antibody coated 96 well ELISA microtiter plate was selected and secured into a holding frame. A 25 μL of calibrator, control and aspirates were pipetted into appropriate wells. All the wells were added with an aliquot of 100 μL working anti-OST HRP conjugate. The plate was then incubated for 2 h at RT. The supernatant was discarded and the wells washed thrice with 400 μL of washing buffer. All the wells were added with an aliquot of 100 μL chromogenic solution within 15 min following the washing step and incubated for 30 min at RT. The plate was then used for an absorbance reading at 450 nm using a microplate reader (Epoch). A standard curve was plotted using optical density values of calibrator against the corresponding OC concentration. The OC concentration in aspirates was extrapolated using linear equation derived from this standard curve.

### Real-time PCR

Gene expression analysis was performed on cells seeded FU scaffolds (n = 3) and cBS (n = 3) on day 1, 7 and 14. RNA was extracted according to the manufacturer’s instruction using an RNeasy Mini Kit (Qiagen, Inc., CA, USA). Later, 1 μg of RNA sample was used to generate cDNA with Qiagen RT2-first strand kit according to the manufacturer’s instructions. Primers for ALP, BMP2, OPN, Runt-related transcription factor 2 (RUNX2), OC, Osteonectin (ON) and GAPDH (housekeeping) were designed using NCBI database (USA) prior to q-PCR (S2 Fig). Primers were validated, optimised and tested for non-template contamination (NTC) before the actual run. Quantitative real-time reverse transcription polymerase chain reaction (qPCR) using designed primers and synthesised cDNA were performed using CFX-96 qPCR machine (Bio-Rad). Primers were used at concentrations ranging between 100 to 500 nm. After an initial denaturation step at 95 ºC for 3 min, the cDNA products were amplified with 35 PCR cycles, comprising a denaturation step at 95 ºC for 30 s, annealing temperature ranging from 50 to 60 ºC and an extension step at 72 ºC for 5 min. Relatively quantified values were analysed using the Bio-Rad CFX manager 2.0. For each cDNA sample, the Ct value of each target sequence was subtracted to the Ct value of the reference gene (GAPDH), to derive ΔCt. The level of expression of each target gene, normalized to housekeeping gene, was then calculated as (1+ Et) ΔCt, where Et is the efficiency of amplification of the target sequence.

## Results and discussion

### hBMSCs characterization

**Fig 1:**
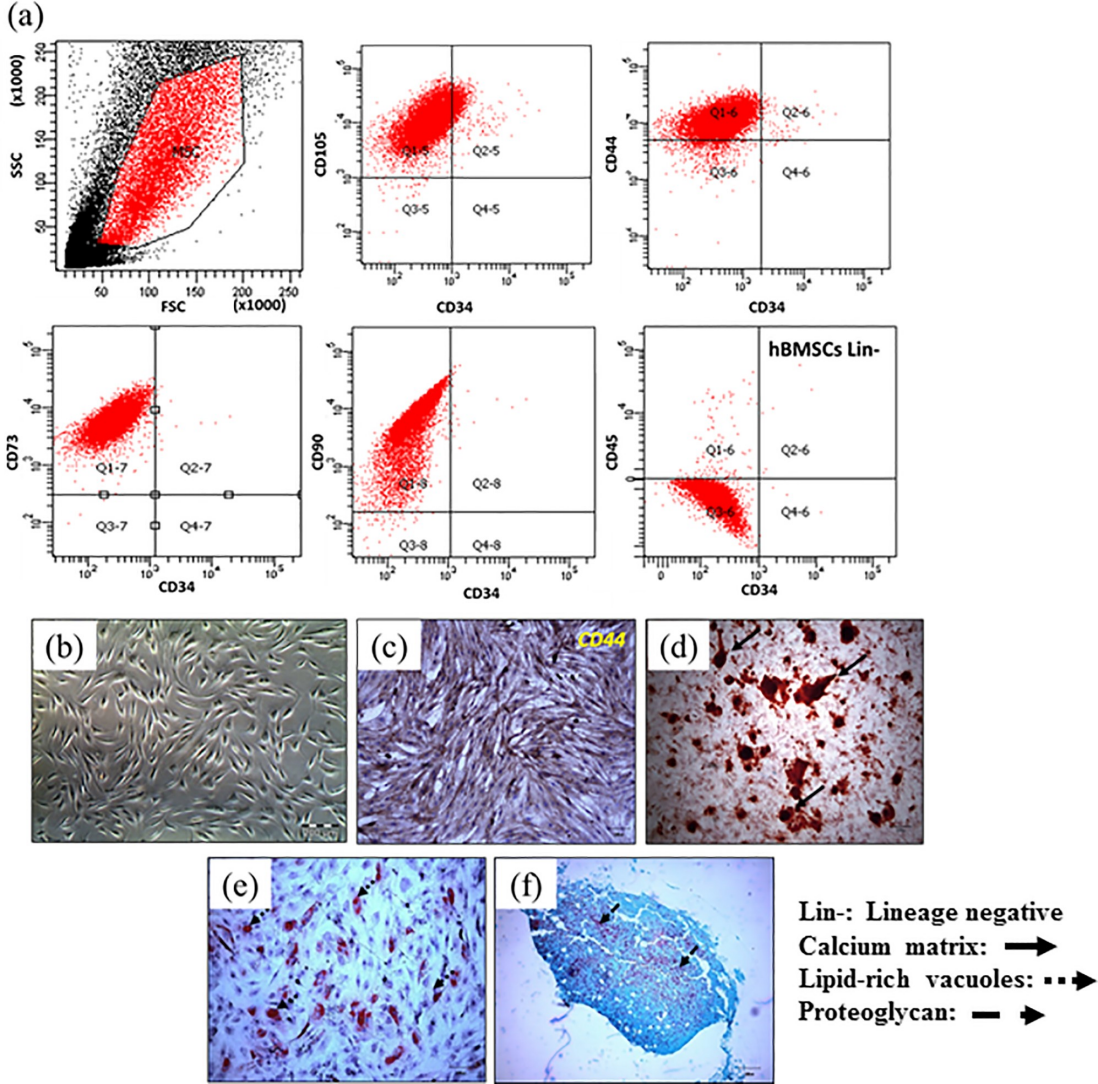
The characterisation of human bone marrow derived mesenchymal stromal cells (hBMSCs). (a) Surface receptor analysis (positive: CD105, CD44, CD73 and CD90 and negative: CD34 & CD45) using flow cytometry, (b) Plastic adherent and fibroblastic-like morphology, (c) CD44 immunocytochemistry (ICC) marker, (d) Alizarin-red S osteogenic staining, (e) Oil-Red O adipogenic staining and (f) Safranin-O chondrogenic staining.

A set of standard to define human MSCs for laboratory-based scientific investigation was proposed by Mesenchymal and Tissue Stem Cell Committee of the International Society for Cellular Therapy (ISCT) [25]. In the present study, prior to apply the hBMSCs for FU scaffold or cBS cell based analysis, their phenotypic characteristics were confirmed using specific surface receptor and functional commitment markers. The surface receptor availability of CD105 and CD44, CD73 and CD90 from flow cytometry analysis confirms the characteristic of hBMSCs (Fig 1A). The contamination of haematopoietic lineage cells from bone marrow were ruled out, when the cells were negative for CD34 and CD45 staining. The plastic adherent and fibroblastic-like morphology were confirmed from phase contrast images (Fig 1B). Moreover, the ICC positive staining for CD44 marker (Fig 1C) further supports the outcomes from flow cytometry analysis. MSCs have a multi-lineage potential and able to differentiate into osteoblasts, chondrocytes and adipocytes [26]. In the present study, the osteogenic (Fig 1D), adipogenic (Fig 1E) and chondrogenic (Fig 1F) potential of isolated hBMSCs were also evidenced by their tri-lineage commitments.

### Physicochemical characteristics

**Fig 2:**
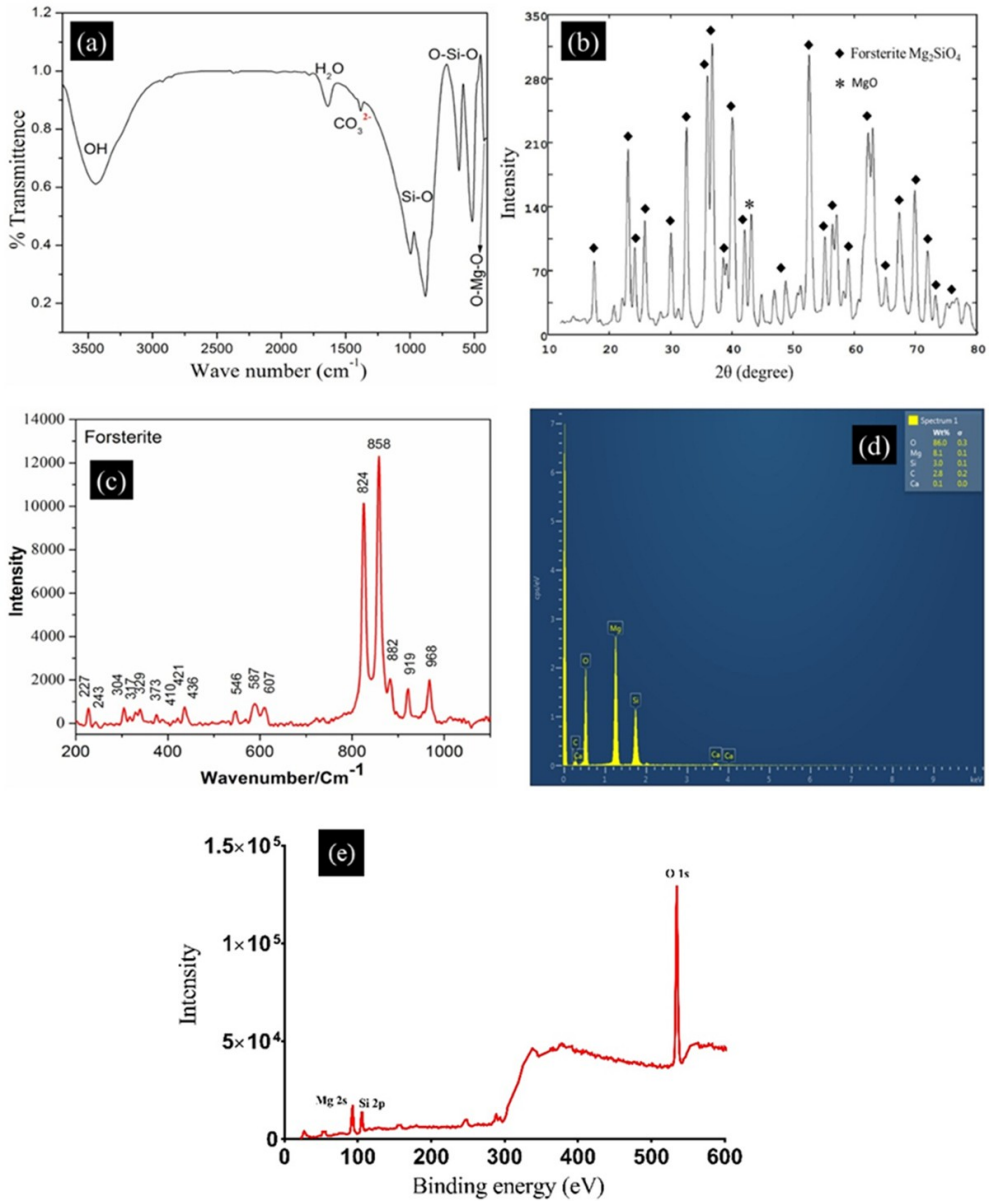
The characterisation of FU. (a) FTIR spectra, (b) XRD pattern, (c) Raman spectra (d) EDX spectra and (e) XPS spectra.

The FTIR spectra of FU scaffold indicate the presence of all fundamental functional groups associated with forsterite (Fig 2A). It was found that the FU scaffold prepared through polymer sponge method comprises of broad moisture band and slight shifting in wavenumbers was also observed. Moreover, the bands corresponding to PVA were also absent in the FTIR spectra indicating the complete elimination of polymer content during thermal treatment. The band at 424 cm^-1^ was attributed to bending modes of O-Mg-O. The peaks at 516 cm^-1^ and 616 cm^-1^ were associated with O-Si-O bending vibrations. The stretching modes of the Si-O band were observed in the range of 879 cm^-1^ to 995 cm^-1^, respectively. The broad band around 3441 cm^-1^ was accounted for OH stretching while sharp peak at 1638 cm^-1^ for H_2_O bending vibrations. The asymmetric stretching of distorted carbonate group was observed at 1383 cm^-1^ leading to splitting in IR band. This was due to absorption of atmospheric carbon dioxide by the material during the reaction process [27, 28]. The XRD pattern shows the presence of FU peaks (Fig 2B). Moreover, no sign of PVA peak in the pattern confirms the existence of FU obtained after polymer sponge method. The MgO peak was found to emerge at 43.08 2θ angle after the thermal treatment at 800 ºC. This observation indicates that FU is highly stable at 800 ºC as similar behaviour of FU was detected during synthesis stage. Thus, the presence of MgO led to shifting in the peaks of FU in XRD pattern. It is very well known fact that the Mg play a key role during skeletal development [29]. Thus, the presence of MgO in FU might assist in increasing the proliferation rate of bone forming cells. Fig 2C shows Raman spectra of FU samples, which was recorded at a wavenumber/cm^-1^ between 200 and 1100. The characteristic peaks observed in Raman spectra were found to have similar wavenumber as that of recently published article [30]. This analysis further strengthens the presence of FU in the scaffolds prepared using polymer sponge method. The EDX spectra (Fig 2D) demonstrate the existence of all elemental peaks corresponding to FU [Magnesium (Mg), Silicon (Si), and Oxygen (O)]. To scrutinise the existence of the defects, XPS was used, as shown in Fig 2E. The binding energy for Mg 2s was observed at 88 eV, which was typical XPS spectrum for FU. The XPS measurements of FU confirmed the presence of Si and O elements as depicted in the survey spectrum with the desired FU stoichiometry having Si:O atom ratio close to 1:3. Thus, this result is in agreement with that of the Raman analysis as mentioned above.

**Fig 3:**
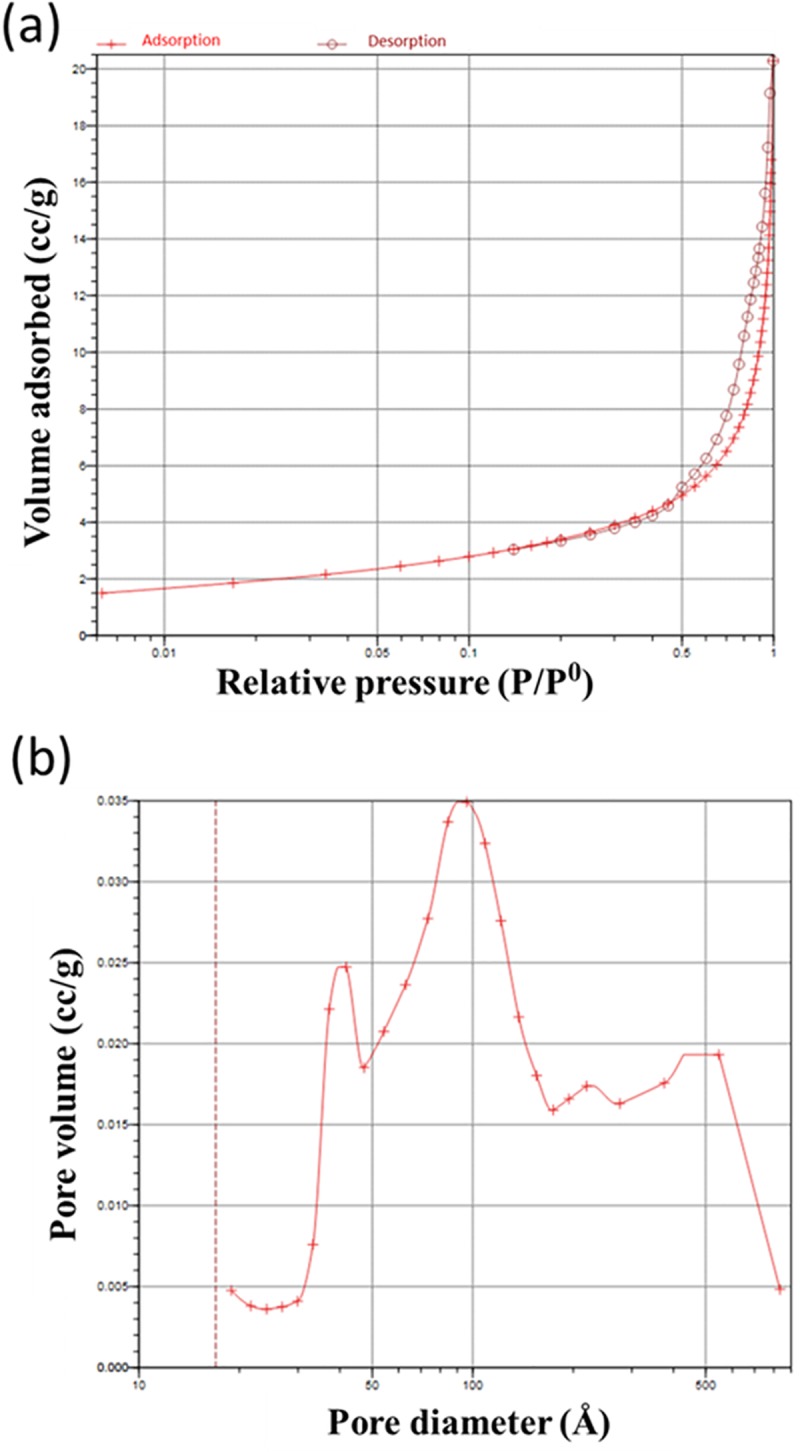
The FU scaffold profile of pores. (a) The Brunauer-Emmett-Teller (BETAutosorb-iQ2) specific surface area of the FU scaffold was measured from the nitrogen adsorption-desorption isotherms and (b) Barret-Joyner-Halenda (BJH) total pore size.

BET analysis (Fig 3A and 3B) of the FU scaffold revealed that this scaffold possessed a typical type IV isotherm with a well-defined presence of mesopores. The FU scaffold showed a surface area of 12.67 m^2^/g and specific pore volume of 0.03 cm^3^/g.

### Biomechanics

**Fig 4:**
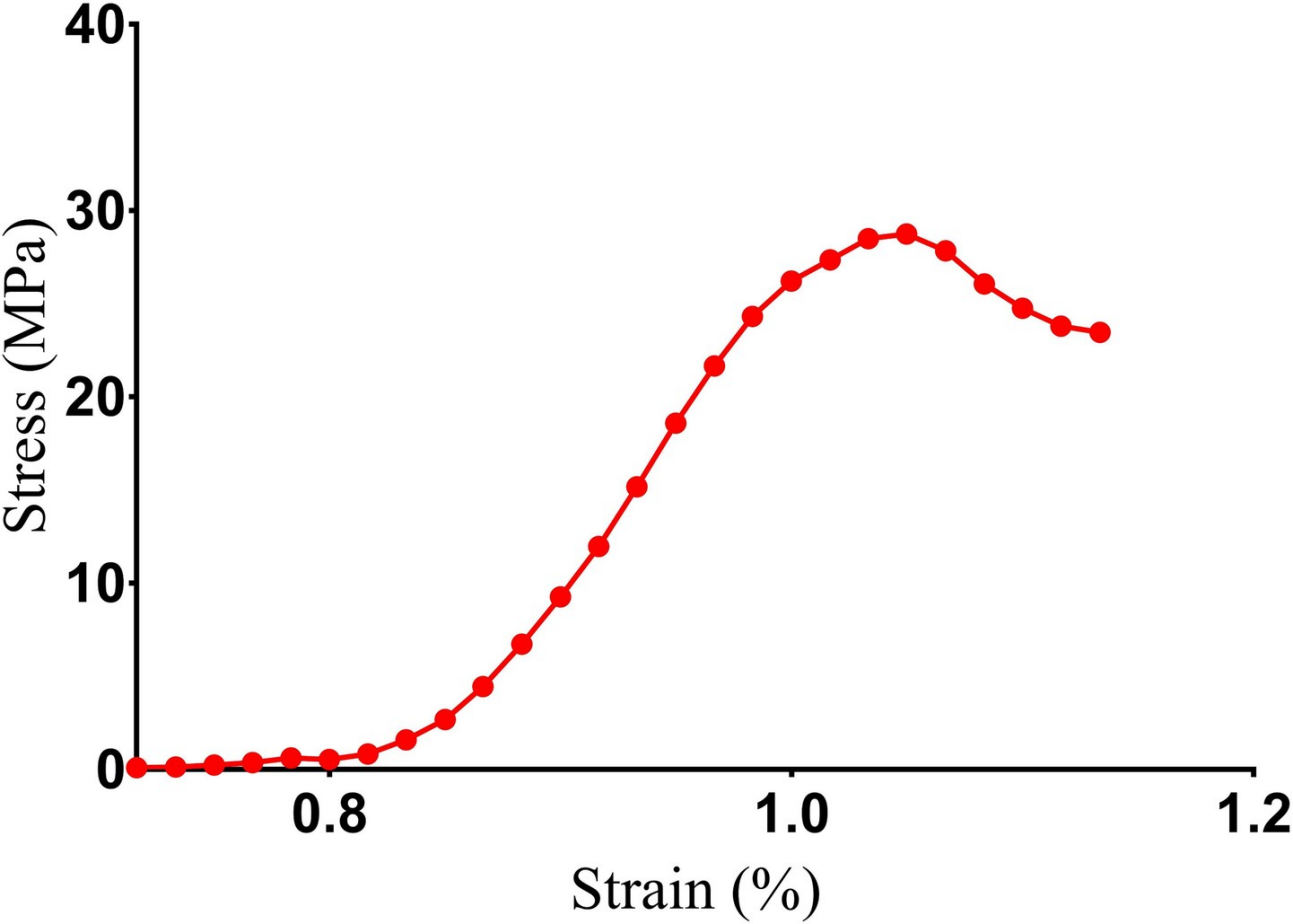
Compressive strength curve. Stress-strain response of FU scaffold sintered at 800 ºC (a representative curve of three individual tests).

Biomechanical properties are crucial characteristics for ceramics, especially when abrasive or grinding action is required. In general, compressive strength is related to crystal structure, bonding, grain size and density, however can be affected by secondary phases, grain boundaries residual stress and impurities [4]. Fig 4 shows a typical stress-strain response following compression of the FU scaffold. The stress increased linearly with an elastic response and failed at a compressive stress of 27.18 ± 13.4 MPa. Although this compressive strength is inferior than that of human cortical bone (~130–290 MPa), yet, it seemed to have a comparable compressive strength with cancellous bone (~2–38 MPa) and superior than other common ceramic based bone substitutes including HA and β-TCP [31]. Thus, we believed that this FU scaffold could be a potential bone substitute mainly for human cancellous bone defects.

### *In vitro* biocompatibility

#### Cell attachment

**Fig 5:**
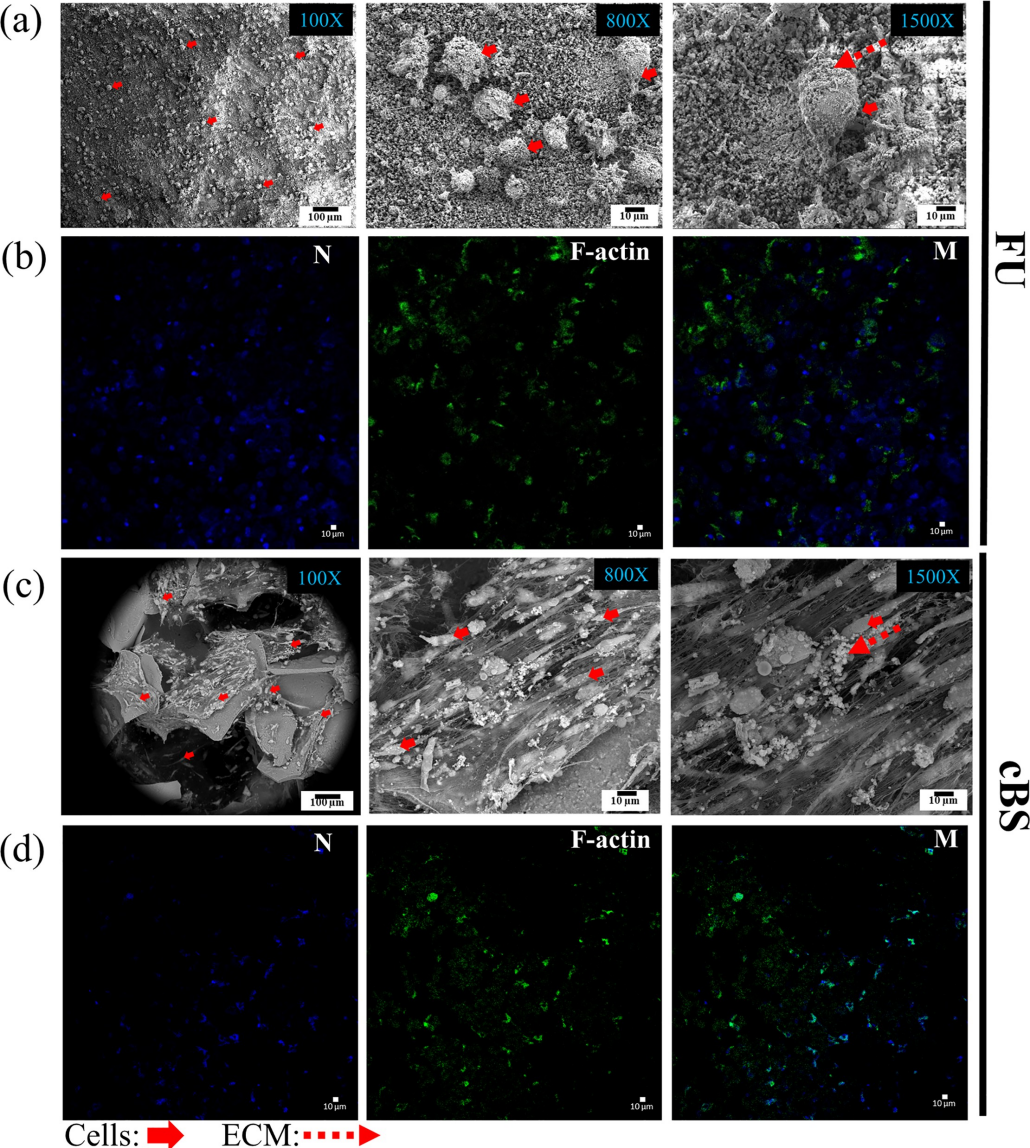
Day 14 hBMSCs attachment on the FU scaffolds and cBS. (a) SEM micrographs and (b) Confocal images of Alexa Fluor 488 phallotoxins F-actin and counterstained with blue fluorescence Hoechst 33342 nucleic acid dye. These images were representative of 3 individual experiments.

The interaction of the hBMSCs within the FU was then examined. The FU scaffolds were seeded with hBMSCs and cell attachment was evaluated using SEM micrographs and CLSM images. Fig 5A-100X and -800X demonstrate that the hBMSCs on the FU scaffold on day 14 extensively colonised the surface of the material and comparable to the cBS (Fig 5C-100X and -800X). This finding noticeably suggests that the chemical composition of FU scaffold was biocompatible and allowed cell attachment. The micrograph at 1500X magnification for both FU and cBS (Fig 5A & 5C) provides an evidence that the cells started secreting extracellular (ECM) components, which modulate the maintenance, proliferation, self-renewal and differentiation of hBMSCs [32]. These ECM components seemed to be corresponding to osteogenic mineral composition as the cells on day 14 could have been committed into a mid-phase of osteoblast lineage. Similar finding was also observed, when hMSCs seeded on poly-L-lactide/hydroxyapatite/collage (PLLA/HA/Col) scaffold showed mineral deposition on the surface of osteoblast-like cells on day 15 of culture duration [33]. The cell-material interaction is referred as a complex bi-directional and dynamic process that appeared to be a natural interaction of cells with ECM matrix. In the present study, the F-actin analysis on hBMSCs seeded on FU scaffold confirms that these cells underwent actin cytoskeleton remodelling to interact with FU (Fig 5B). This finding was comparable to cBS (Fig 5D). This phenomenon can only be noticeable when the surface chemistry of the material is favourable for an optimal cell attachment [34].

### Cell viability and proliferation

**Fig 6:**
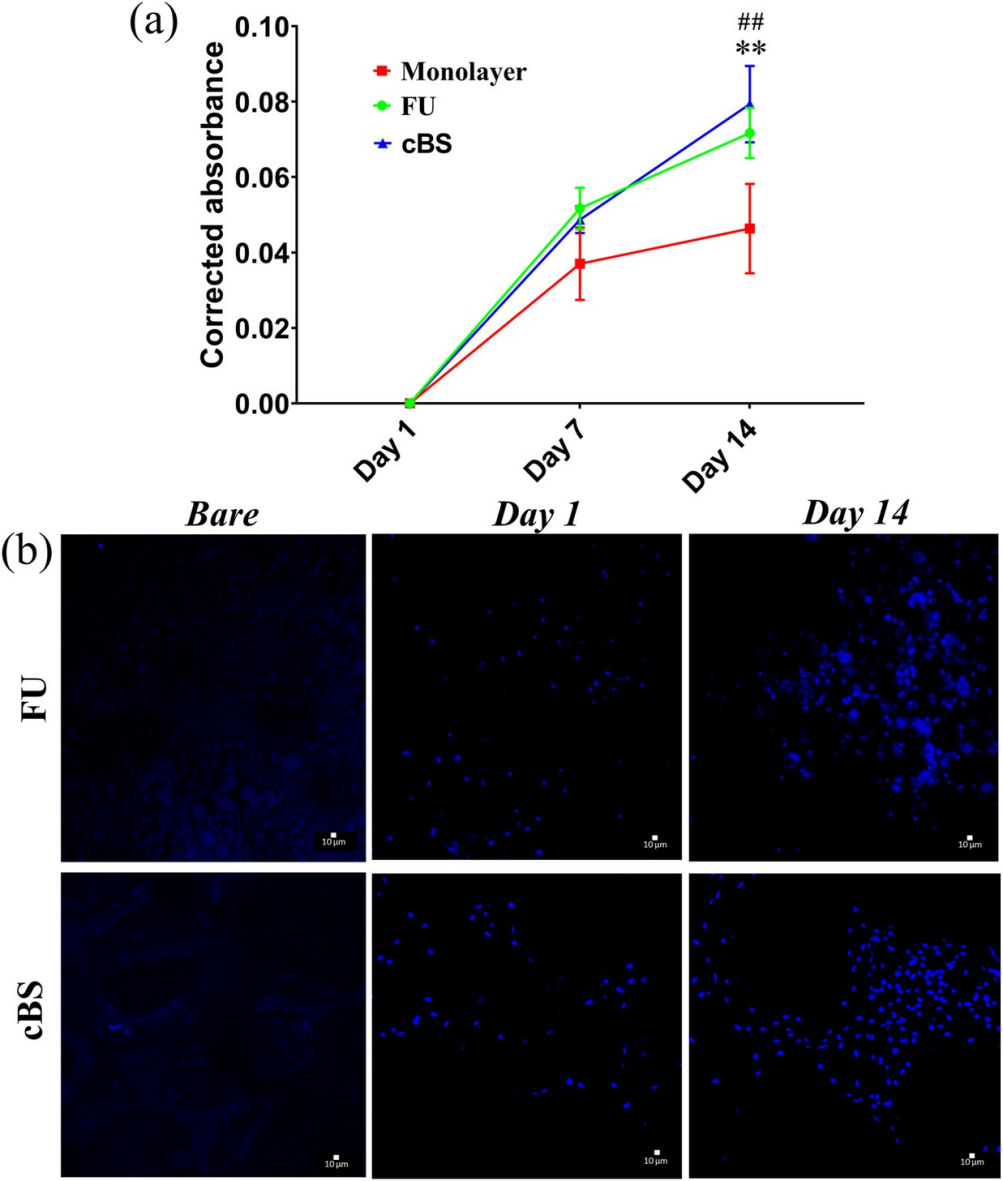
Viability/Proliferation of hBMSCs. (a) Presto blue cell viability measurement of cells seeded on FU scaffold, cBS and monolayer (baseline control) on day 1, 7 and 14, (b) Hoechst 33342 cell-permanent nuclear staining CLSM images of bare FU scaffold/cBS (background control) and cells seeded FU scaffold and cBS on day 1 and 14. (Mann-Whitney U test: cell seeded FU scaffold Vs. monolayer, **p<0.01 and cell seeded cBS Vs. monolayer, ^##^*p*<0.01).

A material engineered for orthopaedic application should maintain cell viability and promote proliferation [23]. The PB cell viability assay demonstrates that the FU scaffold maintained the viability of hBMSCs throughout the culture duration (Fig 6A). In fact, FU scaffold seeded cells were shown a proliferation trend as similar to cBS and monolayer culture. Although cells that grown on both surfaces implicated into a significant cell doublings on day 7 (*p*<0.01) and 14 (*p*<0.01), when compared with day 1, FU scaffold seeded cells showed a 1.5-fold (*p* = 0.003) increase in cell proliferation on day 14, when compared with monolayer. However, this difference was not observed when FU scaffold compared with cBS. Moreover, the confocal analysis on Hoechst 33342 nucleic acid stain further evidenced an increase in cell number of cells seeded on FU scaffolds on day 14, when compared with day 1 (Fig 6B).This finding summarising the fact that FU scaffold has a characteristic to provide an optimal substrate for cell growth and complies a prerequisite standard for commercial bone substitutes (S1 Table).

### *In vitro* cell differentiation

#### Osteogenic protein release

**Fig 7:**
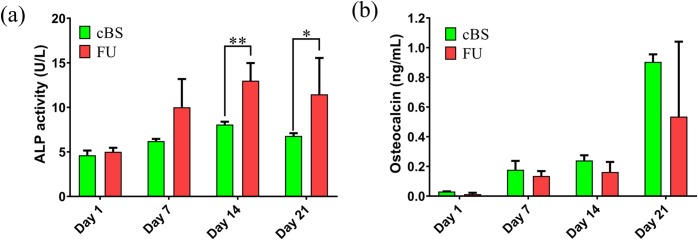
The ELISA assays on media harvested from culture of hBMSCs seeded FU scaffolds and cBS on day 1, 7, 14 and 21. (a) Alkaline phosphatase (ALP) and (b) Osteocalcin (OC). (Mann-Whitney U test: *p<0.05 and **p<0.01).

Our previous study demonstrated bone-like appetite formation on FU scaffold when immersed in simulated body fluid suggesting an excellent bioactivity properties of this silicate [21]. Osteogenic differentiation and mineralized matrix secretion involve the expression of various specific genes and proteins to guide this mechanism [35]. To explore the role of FU in hBMSCs commitment and differentiation into osteogenic lineage, a study was, therefore, conducted on hBMSCs seeded FU scaffold and compared with hBMSCs seeded on cBS using several osteogenic related markers at different time points. ALP and OC are time-dependent osteoblastic markers, which indicate the osteogenic differentiation pattern of hBMSCs [23]. Interestingly, the ALP enzyme activity on day 7, 14, and 21 was 2-, 2.6-and 2.3-fold greater, respectively, when compared with day 1. However, a significant increase was only observed on day 14 (*p* = 0.004) and 21 (*p* = 0.017) (Fig 7A). Similarly, on day 14 and 21, the ALP expression of differentiating hBMSCs on FU scaffold was 1.6-fold (*p* = 0.002) and 1.7-fold (*p* = 0.04), respectively, increased when compared with cBS. This observation evidenced the fact that the osteogenic differentiation process in hBMSCs seeded onto FU scaffold was undertaken in timely manner and comparable with a commercial bone substitute (S2 Table).

OC is a bone matrix protein that occupies 20% of total non-collagenous matrix of bone. This protein is produced and secreted primarily by osteoblasts in the process of mineralization [36]. OC is therefore well known as late stage osteoblastic differentiation protein and it is also considered osteoblastic-specific protein [4]. In the present study, the OC secretion at different time points was demonstrated using OC Elisa assay (Fig 7B). It was found that the hBMSCs seeded onto FU scaffold were shown a significant increase in OC secretion on day 21 (0.534 ng/mL, *p* = 0.035) when compared with day 1 (0.013 ng/mL). However, no significant differences in OC secretion were observed on day 7 (0.136 ng/mL, *p* = 0.837) and 14 (0.163 ng/mL, *p* = 0.748) when compared with day 1. Similar pattern of OC expression as seen in the hBMSCs seeded onto FU scaffold was also observed in hBMSCs seeded onto cBS. This expression in hBMSCs-cBS was 29-fold greater on day 21 (*p* = 0.001) when compared with day 1. This finding allows to speculate that the hBMSCs seeded onto FU scaffold underwent osteogenic differentiation as comparable to cBS, corresponding to intact OC release in culture media (S2 Table). This finding was further supported by a parallel observation reported by Nakamura and colleagues. They found an increase in OC in rat bone marrow derived MSCs that were seeded on HA ceramic on day 16 of the observation [37].

### Osteogenic intra-and extra-cellular protein expression

**Fig 8:**
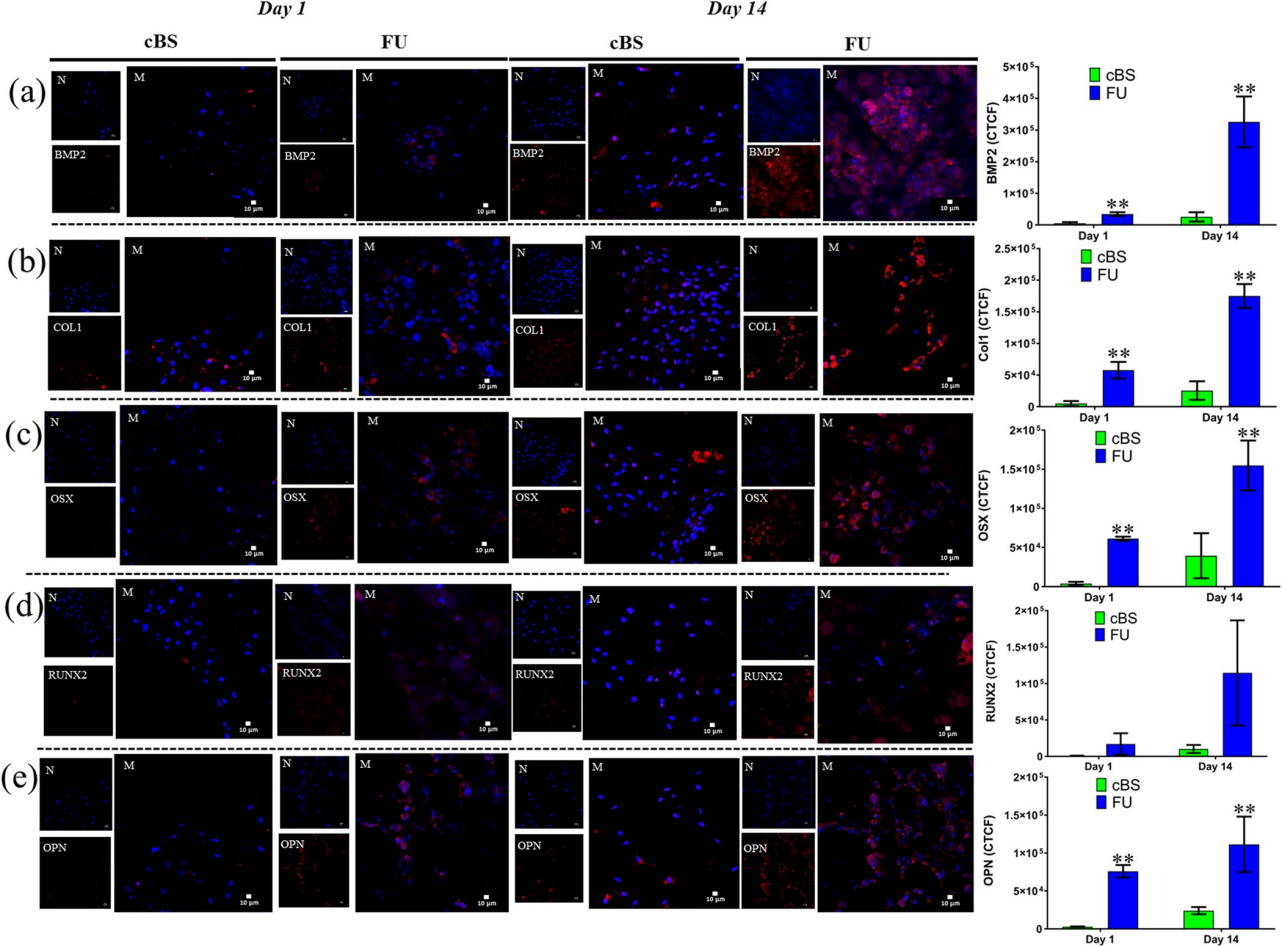
CLSM images of intra- and/or extra-cellular proteins expressed by hBMSCs seeded onto FU scaffold and cBS on day 1 and 14. (a) Bone morphogenetic protein-2 (BMP2), (b) Type-1 collagen (Col1), (c) Osterix (OSX), (d) Runt-related transcription factor2 (RUNX2) and (e) Osteopontin (OPN). (Mann-Whitney U test: **p<0.01). CTCF: Corrected total cell fluorescence.

BMP2 is a growth factor that commonly used to promote osteogenic differentiation during bone repair [38]. In the current study, an increased BMP2 protein secretion was obvious from the confocal images on day 14 compared to day 1. The CTCF intensity of BMP2 on day 14 samples was 9-fold (*p* = 0.003) greater than that of day 1 (Fig 8A) samples. In addition, a significant increase of BMP2 was also observed in hBMSCs seeded onto FU when compared with cBS on day 1 and 14 (S3 Table). This finding confirms the fact that the hBMSCs seeded on FU scaffold could have committed to pre-osteoblastic lineage [39].

Type-I collagen (Col1) is one of the major matrix components of the bone [40]. During differentiation, MSCs first give rise to osteoblasts, which progressively express markers concomitant to a mature phenotype including Col1 [41]. Day 14 confocal images indicate an overwhelming Col1 secretion on differentiating hBMSCs. The CTCF intensity confirms that the Col1 protein in hBMSCs seeded onto FU was higher on day 14 (*p* = 0.001) when compared with Day 1(Fig 8B). A significant expression of Col1 in hBMSCs seeded onto FU scaffold on day 1 (5-fold, *p* = 0.012) and 14 (11-fold, *p* = 0.001) was also observed when compared with cBS (S3 Table). In previous studies, collagen was used either as culture dish coating or in 3D gel format to induce MSCs osteogenic differentiation [42]. In the present study, the Col1 secretion without exogenous type-1 collagen molecules in media indicating the hBMSCs could be well adapted on the FU scaffold surface and deposited Col1 extracellularly to create a microenvironment favourable for osteogenic differentiation and matrix mineralisation [43]. This notion was well supported by our SEM micrographs of hBMSCs with noticeable ECM on the cellular periphery and surroundings.

Osterix (OSX) is a bone-related transcription factor that orchestrates downstream of RUNX2, which regulates both growth and differentiation in osteoblast [44]. The number of cells secreting OSX was significantly increased from day 1 to Day 14 on both FU and cBS (*p*<0.05). These significant increases were also present when compared between FU scaffold and cBS (*p*<0.05) (Fig 8C). OSX secretion in hBMSCs corroborates with our BMP2 outcome and indicates a successful osteogenic pathway commitment in MSCs. Conversely, the expression of RUNX2 either between days or materials was comparable (S3 Table). This can be explained by the fact that the hBMSCs commitment to osteoblast lineage could still be controlled by other protein such as Dlx5 as suggested by Hyun-Mo and colleagues in their MSCs osteogenic pathway analysis [45].

Osteopontin (OPN) is another ECM protein that can be found in osteogenic cells differentiating from MSCs. This non-collagenous bone matrix protein mainly involves in bridging between cells and minerals in bone [46]. In the present study, OPN matrix secretion was 2-fold and 8-fold greater on day 14 when compared with day 1 in hBMSCs seeded onto FU scaffold and cBS, respectively (Fig 8E). However, this increase was not significant in both material groups (S3 Table). This can be explained by a reason that an extreme OPN could increase the osteoclast activity, which causes a bone resorption. This because the RGD sequence in OPN can also crosstalk with integrin from the macrophages and translocate NFATc1 to induce osteoclast differentiation [47, 48]. Therefore, it was speculated that the OPN protein expression that was maintained in suboptimal level while MSCs differentiation into osteogenic lineage could be due to the reason of OPN requirement to maintain a basic osteogenic physiological function and osteoclastic activity for bone remodelling. Interestingly, the OPN expression in hBMSCs seeded onto FU scaffold on day 1(26-fold, *p* = 0.001) and 14 (5-fold, *p* = 0.001) was significantly greater when compared with cBS. This finding supports the notion that the FU scaffold has potential to induce osteogenic differentiation in comparable with a commercial bone substitute.

### *In vitro* gene expression

**Fig 9:**
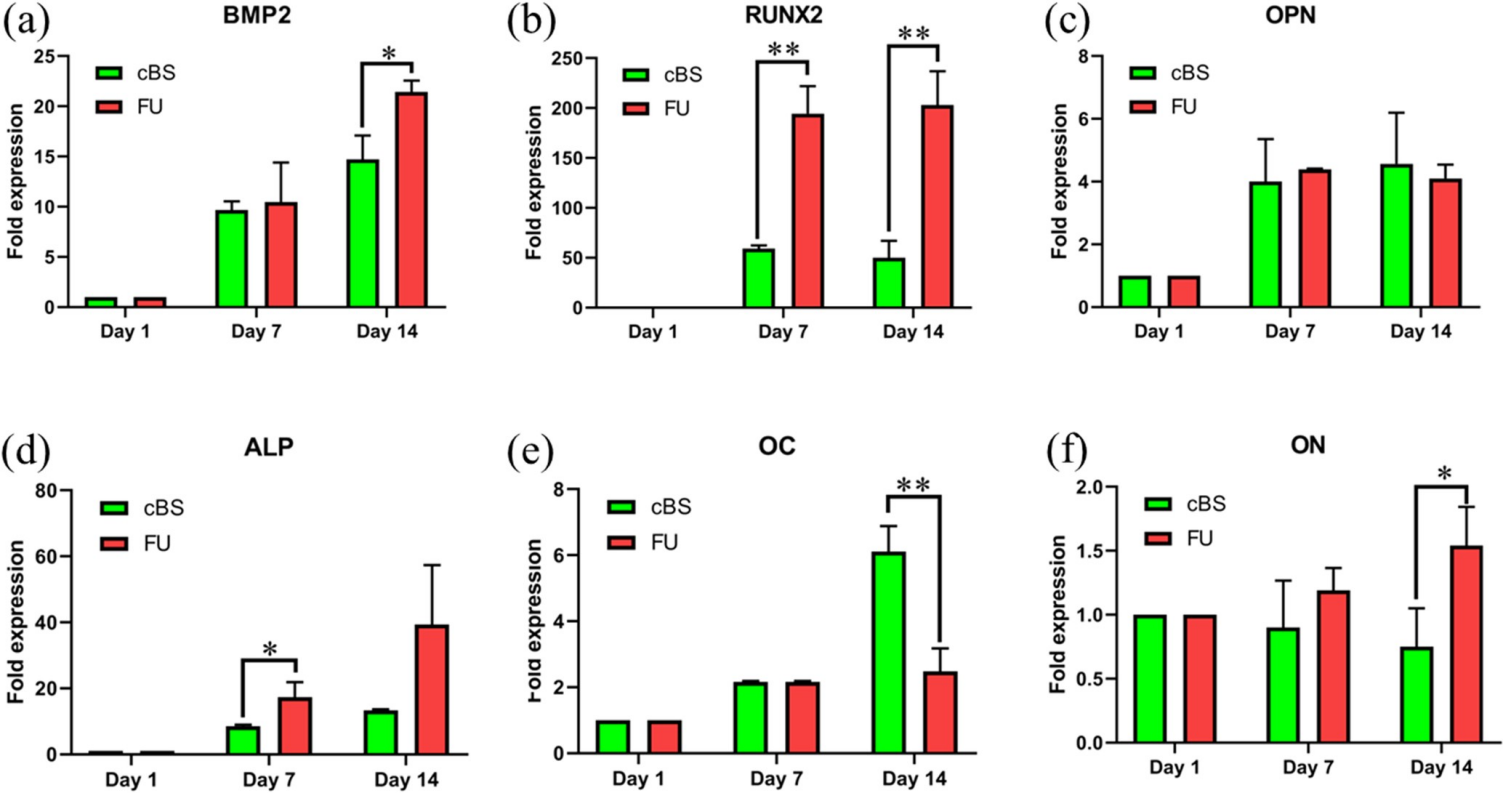
Quantitative gene expression during the differentiation process from day 1 to day14 of the hBMSCs-seeded on FU scaffold and cBS. (a) BMP2, (b) RUNX2, (c) OPN, (d) ALP, (e) OC and (f) Osteonectin (ON). (Kruskal-Wallis test: *p<0.05 and **p<0.01).

The Fig 9 shows osteogenic related gene expression in hBMSCs seeded on FU scaffolds and cBS on day 1, 7 and 14 (S4 Table). The BMP2 fold-increase in hBMSCs seeded on FU scaffolds on day 7 (15-fold, *p* = 0.037) and 14 (33-fold, *p* = 0.006) was significantly higher when compared with day 1 (Fig 9A).This pattern of BMP2 gene expression was also comparable in hBMSCs seeded on cBS. In addition, BMP2 gene expression in hBMSCs seeded on FU scaffold was 1.5-fold (*p* = 0.02) significantly higher when compared with cBS. The BMP2 gene expression outcome was well correlated with our confocal data for BMP2 protein expression. This finding clearly suggests the notion that the FU scaffold has potential to induce hMSC differentiation into osteogenic lineage. Moreover, the Mg^2+^ ions as an ultimate component in FU could be one of the inducers for hBMSCs commitment into osteogenic lineage. This scenario was also reported in a predated study where the BMP2 expression on hBMSCs was markedly increased when seeded on magnesium phosphate ceramic [49]. This could be due to the capacity of Mg^2+^ ions in promoting hMSCs differentiation into osteogenic lineage through activating Wnt signalling pathway [50].

It was observed that hBMSCs seeded onto FU scaffold showed a 186-fold (*p* = 0.010) and 194-fold (*p* = 0.008) increase in RUNX2 on day 7 and 14, respectively (Fig 9B). Similarly, about 3-fold (*p*<0.01) and 4-fold (*p*<0.01) of RUNX2 expression was found in hBMSCs seeded onto FU scaffold when compared with cBS. Although these significant increases were not observed in protein level between day 1 and 14 or FU scaffold and cBS of CLSM images, the considerable increase in gene expression level evidenced that the hBMSCs might have initiated the molecular level of osteogenic lineage commitment. Similar outcome was also observed in a study when hMSCs seeded on coragraf incorporated with platelet-derived growth factor-BB (PDGF-BB) [51]. This growth factor seemed to be involved in inducing hMSCs differentiation into osteogenic lineage through upregulating RUNX2 transcription factor. Interestingly, in the present study, RUNX2 expression in hBMSCs seeded on FU scaffold was overwhelming without involving any osteogenic inducing growth factors. This finding suggests that the FU could have an inherent characteristic to support hBMSCs osteogenic differentiation.

In bone remodelling, OPN appears to be an important marker to indicate the osteogenic capacity of MSCs in early stage of osteogenesis [52]. In the current study, the OPN gene expression was significantly greater on day 7 and day14 when compared with day 1 (Fig 9C). However, there was no difference found when compared between materials. In another study, it was reported that the osteogenic induced bone marrow MSCs expressed 4-fold of OPN on day 14 when compared with day 0 and 7 [53]. However, our results confirmed that the OPN expression was kicked up on day 7 onward suggesting an early osteoblastic commitment of hBMSCs.

The pattern of ALP and OC gene expression (Fig 9D & 9E) in hBMSCs seeded onto FU and cBS was comparable with ALP and OC protein expression. In fact the significant ALP gene expression in hBMSCs seeded onto FU scaffold on day 7 (2-fold, *p* = 0.02) was parallel with the protein expression on the same time point. This finding clearly confirms the notion that the total ALP expressed on molecular level on day 7 was effectively undertaken post-translational modification as a functional protein to support osteogenic characteristic of hBMSCs.

Osteonectin (ON) is a non-collagenous component of the ECM that can be primary found in bone. It is also considered as bone-specific because of its biochemical properties, such as a marker related to osteoblastic functional differentiation [54]. When hBMSCs seeded on FU scaffold, it showed a significant increase in ON level primary on day 14 (*p* = 0.03) when compared with cBS (Fig 9F). A previous study reported that ON expression was observed in the petri dish during osteogenic differentiation with or without prerequisite ECM coating. When the stromal cells were cultivated in the petri dish in the absent of ECM, the ON expression was relatively dropped during the early stages of differentiation, but tend to increase after the addition of the osteogenic growth factors [55]. In the present study, the significant expression of ON in hBMSCs seeded on FU scaffold indicates its inherent competency to support osteogenic differentiation of this stromal cell without any osteogenic supplements.

## Conclusion

In conclusion, the physicochemical, surface chemistry and biomechanical profile of FU was not altered after the application of polymer sponge scaffold fabrication method. In biological perspective, FU scaffold supports hBMSCs attachment and proliferation and comparable to a commercial bone substitutes. Furthermore, FU scaffold induces osteogenic commitment of hBMSCs. These findings indicate that FU scaffold is biocompatible as well as can facilitate osteogenic differentiation of hBMSCs *in vitro*, therefore, *in vivo* animal study on FU scaffold is highly recommended prior to be tested in clinical trials.

## Supporting information

S1 Fig Isotype controls. The isotype controls for CD34, CD105, CD73, CD90, CD45 and CD44 were used to differentiate non-specific background signal from specific antibody signal.
(TIF)

S2 Fig Sequence of forward and reverse primers. Primers for ALP, BMP2, OPN, RUNX2, OC, ON and GAPDH (housekeeping) were designed using NCBI database for qPCR analysis.
(TIF)

S1 Table Cell viability. Presto blue cell viability measurement of hBMSCs seeded on FU scaffold, cBS and monolayer (baseline control) on day 1, 7 and 14. The presto blue absorbance readings on day 7 and 14 of FU scaffold, cBS and monolayer were corrected by subtracting the day 1 presto blue absorbance readings of the respective groups.
(PDF)

S2 Table Expression of osteogenic proteins. The Alkaline phosphatase (ALP) and Osteocalcin (OC) concentration in media harvested from culture of hBMSCs seeded FU scaffolds and cBS were measured on day 1, 7, 14 and 21 using ELISA technique.
(PDF)

S3 Table Osteogenic differentiation protein analysis using confocal. The intra- and/or extra-cellular proteins expressed by hBMSCs seeded onto FU scaffold and cBS on day 1 and 14 were imaged using confocal laser scanning microscopy (CLSM) and the images were analysed using Image-J analysis software. The data were presented as corrected total cell fluorescence (CTCF).
(PDF)

S4 Table Expression of osteogenic genes. Quantitative gene expression of osteogenic genes during the differentiation process from day 1 to day 14 of the hBMSCs seeded on FU scaffold and cBS was studied using a qPCR technique. The gene expression outcomes were normalised with GAPDH (housekeeping gene) and fold-change for day 7 and 14 calculated by using day 1 gene expression as a baseline.
(PDF)
