# Supplementary figures and images for: The physicochemical and biomechanical profile of forsterite and its osteogenic potential of mesenchymal stromal cells
Source: PLoS One. 2019 Mar 27;14(3):e0214212. doi: 10.1371/journal.pone.0214212 (PMC6436741; doi:10.1371/journal.pone.0214212)

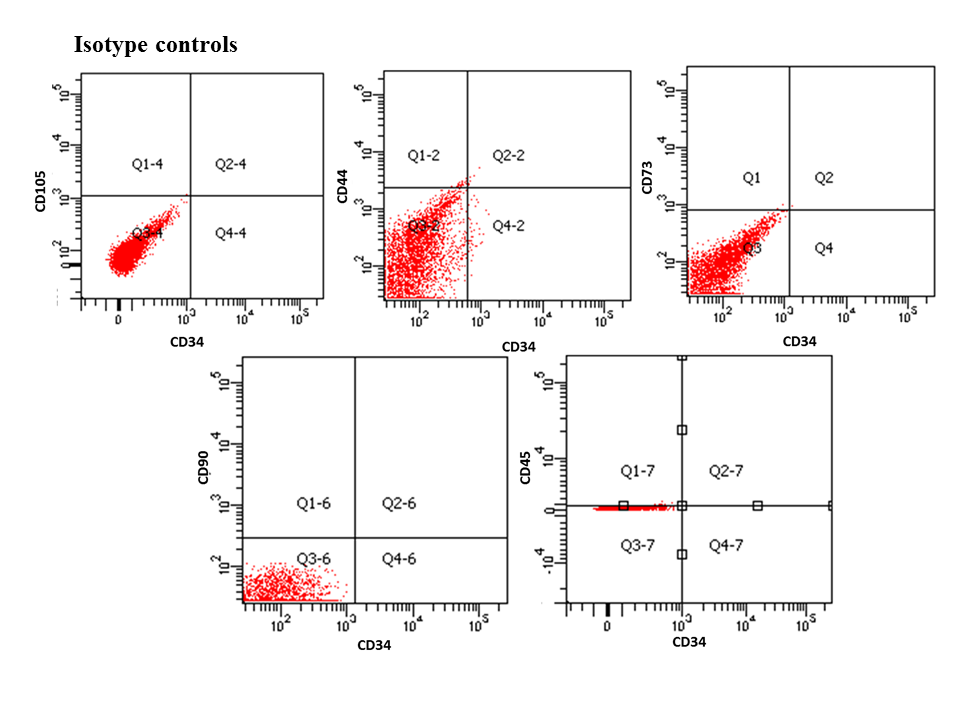

Supplement: S1 Fig — The isotype controls for CD34, CD105, CD73, CD90, CD45 and CD44 were used to differentiate non-specific background signal from specific antibody signal. (TIF) [file pone.0214212.s001.tif]

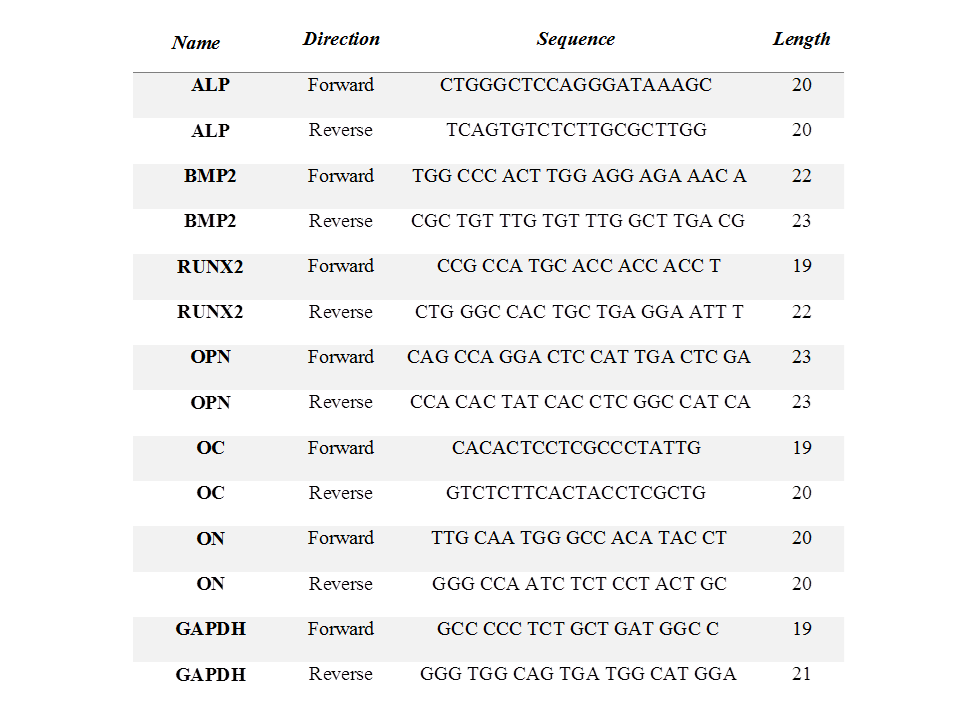

Supplement: S2 Fig — Primers for ALP, BMP2, OPN, RUNX2, OC, ON and GAPDH (housekeeping) were designed using NCBI database for qPCR analysis. (TIF) [file pone.0214212.s002.tif]
