# Supplementary material for: The physicochemical and biomechanical profile of forsterite and its osteogenic potential of mesenchymal stromal cells
Source: PLoS One. 2019 Mar 27;14(3):e0214212. doi: 10.1371/journal.pone.0214212 (PMC6436741; doi:10.1371/journal.pone.0214212)
Supplement: S1 Table — Presto blue cell viability measurement of hBMSCs seeded on FU scaffold, cBS and monolayer (baseline control) on day 1, 7 and 14. The presto blue absorbance readings on day 7 and 14 of FU scaffold, cBS and monolayer were corrected by subtracting the day 1 presto blue absorbance readings of the respective groups. (PDF) [file pone.0214212.s003.pdf]

Day	Samples	Monolayer	FU	cBS
1	1	0	0	0
	2	0	0	0
	3	0	0	0
7	1	0.035	0.055	0.065
	2	0.028	0.054	0.052
	3	0.047	0.045	0.069
14	1	0.053	0.076	0.083
	2	0.054	0.064	0.091
	3	0.033	0.075	0.084
