# Supplementary material for: The physicochemical and biomechanical profile of forsterite and its osteogenic potential of mesenchymal stromal cells
Source: PLoS One. 2019 Mar 27;14(3):e0214212. doi: 10.1371/journal.pone.0214212 (PMC6436741; doi:10.1371/journal.pone.0214212)
Supplement: S2 Table — The Alkaline phosphatase (ALP) and Osteocalcin (OC) concentration in media harvested from culture of hBMSCs seeded FU scaffolds and cBS were measured on day 1, 7, 14 and 21 using ELISA technique. (PDF) [file pone.0214212.s004.pdf]

Day	ALP activity (U/L)	
	cBS	FU
1	4.5	5.4
	4.1	4.8
	4.5	5.4
	5.4	4.5
7	6.2	11.7
	6.5	10.7
	5.9	12.3
	6.2	5.4
14	8.3	12.4
	8.1	15.8
	7.6	12.5
	8.3	11.2
21	7.2	13.0
	6.4	5.4
	6.7	13.1
	6.8	14.3

**Day**	**OC activity (ng/mL)**	
	**cBS**	**FU**
1	0.03	0.02
	0.03	0.00
	0.03	0.01
	0.03	0.01
7	0.15	0.11
	0.24	0.12
	0.21	0.13
	0.11	0.18
14	0.29	0.14
	0.24	0.15
	0.22	0.26
	0.21	0.10
21	0.83	0.85
	0.94	1.08
	0.94	0.07
	0.90	0.14
