# Supplementary material for: The physicochemical and biomechanical profile of forsterite and its osteogenic potential of mesenchymal stromal cells
Source: PLoS One. 2019 Mar 27;14(3):e0214212. doi: 10.1371/journal.pone.0214212 (PMC6436741; doi:10.1371/journal.pone.0214212)
Supplement: S3 Table — The intra- and/or extra-cellular proteins expressed by hBMSCs seeded onto FU scaffold and cBS on day 1 and 14 were imaged using confocal laser scanning microscopy (CLSM) and the images were analysed using Image-J analysis software. The data were presented as corrected total cell fluorescence (CTCF). (PDF) [file pone.0214212.s005.pdf]

BMP2					
Day	BMP2	Area	Mean	intDen	CTCF
1	Control (Background)	10355	0	584	
1	Fu 1	11730	3	35654	34997
1	Fu 2	12720	3	40561	39849
1	Fu 3	9476	3	28806	28275
14	Control (Background)	11220	0	4801	
14	Fu 1	17514	23	403408	395912
14	Fu 2	17399	20	351130	343683
14	Fu 3	14742	17	244294	237984
Type I-Collagen					
Day	Col1	Area	Mean	intDen	
1	Control (Background)	10712	0	15	
1	Fu 1	10712	6	69579	69568
1	Fu 2	13455	4	60006	59993
1	Fu 3	13455	3	43738	43725
14	Control (Background)	13447	0	535	
14	Fu 1	13447	15	196508	195970
14	Fu 2	13447	13	169974	169436
14	Fu 3	14880	11	160304	159709
Osterix					
Day	OSX	Area	Mean	intDen	
1	Control (Background)	16764	0	2	
1	Fu 1	16764	4	60790	60800
1	Fu 2	16764	4	64127	64100
1	Fu 3	16764	4	59126	59100
14	Control (Background)	16698	0	3	
14	Fu 1	16698	11	186221	186218
14	Fu 2	16698	9	155858	155855
14	Fu 3	16698	7	122349	122346
RUNX2					
Day	RUNX2	Area	Mean	intDen	
1	Control (Background)	12535	3	40126	
1	Fu 1	12535	6	71644	31519
1	Fu 2	12535	3	42195	2070
1	Fu 3	12535	5	57992	17867
14	Control (Background)	12852	2	26769	
14	Fu 1	12852	17	224055	197284
14	Fu 2	12852	7	92046	65275
14	Fu 3	12852	8	108069	81298
Osteopontin					
Day	OPN	Area	Mean	intDen	
1	Control (Background)	11000	0	325	
1	Fu 1	11000	7	72657	72333
1	Fu 2	11000	6	70565	70241
1	Fu 3	11000	8	85769	85445
14	Control (Background)	10864	0	1780	

14	Fu 1	10864	16	88110	86328
14	Fu 2	10864	9	155121	153339
14	Fu 3	10864	8	96331	94549

BMP2					
Day	BMP2	Area	Mean	intDen	CTCF
1	Control (Background)	1	0	0	
1	cBS 1	1	3	9576	9575
1	cBS 2	1	1	2635	2633
1	cBS 3	1	1	3143	3143
14	Control (Background)	1	0	727	
14	cBS 1	1	15	42519	42516
14	cBS 2	1	6	17167	17164
14	cBS 3	1	6	16929	16926
Type I-Collagen					
Day	Col1	Area	Mean	intDen	
1	Control (Background)	1	0	0	
1	cBS 1	1	10	26605	26604
1	cBS 2	1	2	4590	4589
1	cBS 3	1	2	4165	4164
14	Control (Background)	1	0	1076	
14	cBS 1	1	7	19238	19238
14	cBS 2	1	4	11682	11680
14	cBS 3	1	6	15149	15145
Osterix					
Day	OSX	Area	Mean	intDen	
1	Control (Background)	1	0	16	
1	cBS 1	1	2	6448	6448
1	cBS 2	1	1	2432	2431
1	cBS 3	1	1	3118	3118
14	Control (Background)	1	0	85	
14	cBS 1	1	7	19040	19038
14	cBS 2	1	10	27030	27026
14	cBS 3	1	26	72420	72415
RUNX2					
Day	RUNX2	Area	Mean	intDen	
1	Control (Background)	1	0	0	
1	cBS 1	1	0	1150	1148
1	cBS 2	1	0	1018	1018
1	cBS 3	1	0	939	939
14	Control (Background)	1	0	255	
14	cBS 1	1	6	16575	16574
14	cBS 2	1	2	6715	6712
14	cBS 3	1	3	7480	7480
Osteopontin					
Day	OPN	Area	Mean	intDen	

1	Control (Background)	1	0	29	
1	cBS 1	1	1	2469	2469
1	cBS 2	1	1	3012	3011
1	cBS 3	1	1	3151	3151
14	Control (Background)	1	0	354	
14	cBS 1	1	10	26371	26371
14	cBS 2	1	7	18569	18564
14	cBS 3	1	10	27206	27202
