# Supplementary material for: The physicochemical and biomechanical profile of forsterite and its osteogenic potential of mesenchymal stromal cells
Source: PLoS One. 2019 Mar 27;14(3):e0214212. doi: 10.1371/journal.pone.0214212 (PMC6436741; doi:10.1371/journal.pone.0214212)
Supplement: S4 Table — Quantitative gene expression of osteogenic genes during the differentiation process from day 1 to day 14 of the hBMSCs seeded on FU scaffold and cBS was studied using a qPCR technique. The gene expression outcomes were normalised with GAPDH (housekeeping gene) and fold-change for day 7 and 14 calculated by using day 1 gene expression as a baseline. (PDF) [file pone.0214212.s006.pdf]

	cBS			FU		
**Genes**	**Day 1**	**Day 7**	**Day 14**	**Day 1**	**Day 7**	**Day 14**
**OPN**	1	2.56	4.40	1	4.378	3.841
	1	5.24	6.28	1	4.41	4.61
	1	4.21	3.04	1	4.40	3.85
**BMP2**	1	9.12	16.92	1	8.18	20.24
	1	10.67	15.03	1	15.02	22.52
	1	9.27	12.18	1	8.13	21.53
**OC**	1	7.51	6.92	1	2.12	1.69
	1	7.63	6.03	1	2.18	2.90
	1	6.29	5.38	1	2.17	2.87
**RUNX2**	1	57.81	67.66	1	208.53	163.81
	1	62.82	34.06	1	162.80	225.10
	1	56.90	48.37	1	212.27	220.02
**ON**	1	1.25	0.82	1	0.99	1.19
	1	0.93	1.01	1	1.29	1.76
	1	0.52	0.42	1	1.29	1.67
**ALP**	1	8.12	12.91	1	22.56	50.03
	1	8.31	13.26	1	15.12	18.64
	1	9.01	13.67	1	14.27	49.53
